# Oncocytic papillary renal cell carcinoma (OPRCC): 2 case report and literature review

**DOI:** 10.3389/fonc.2024.1541692

**Published:** 2025-01-31

**Authors:** Yanchen Wang, Lihui Guan, Yaming Liu, Yuxuan Liu, Xiaoyan Guo, Yaofei Sun

**Affiliations:** ^1^ Department of Urology, Weifang People’s Hospital, Shandong Second Medical University, Weifang, Shandong, China; ^2^ Shandong Provincial Key Laboratory for Prevention and Treatment of Urological Diseases in Medicine and Health, Weifang, Shandong, China; ^3^ Department of Urology, Yidu Central Hospital of Weifang City, Weifang, Shandong, China; ^4^ School of Clinical Medicine, Shandong Second Medical University, Weifang, China; ^5^ Department of Nuclear Medicine, Weifang People’s Hospital, Shandong Second Medical University, Weifang, Shandong, China

**Keywords:** PRCC, OPRCC, immunohistochemistry, genetic pathological features, targeted immune drugs

## Abstract

Oncocytic papillary renal cell carcinoma (OPRCC) is a new papillary renal cell carcinoma (OPRCC) added to the 2016 WHO Classification of Tumors of the Urinary and Male Reproductive System. It is a subtype of carcinoma, PRCC, which is very rare and it is very characteristic and interesting. The morphology, immunophenotype, genetic characteristics and prognosis of OPRCC are uncertain, so the diagnosis and post-operative management of OPRCC are challenging. Recently, two patients with OPRCC have been treated in our hospital, both of whom have undergone surgical treatment. The information of these two cases is described as follows, and the literature is further reviewed to share the current diagnosis and treatment characteristics of OPRCC and the research progress after surgery. At the same time, the diagnosis of OPRCC disease is subdivided from PRCC disease, and the diagnosis of OPRCC disease is more precise. To optimize the individualized management of patients with renal cell carcinoma in order to improve the understanding and diagnosis of the disease.

## 1 Introduction

Renal carcinoma is a malignant tumor originating from tubular epithelial cells of the kidney. As the third most common tumor of the urinary system, renal carcinoma ranks the 12th among the most common cancers in the world, accounting for 3% of adult malignant tumors. Among them, clear cell renal cell carcinoma accounts for about 70%, and papillary renal cell carcinoma accounts for about 15%. Although the 5th edition of the WHO Classification of renal cell Carcinoma no longer distinguishes PRCC type I from PRCC type II, from the perspective of clinicians observing the disease progression and prognosis of patients, the distinction between PRCC type I and PRCC type II is very instructive. Papillary renal cell carcinoma type I progresses slowly, whereas papillary renal cell carcinoma type II progresses relatively quickly and has a poor prognosis.

OPRCC, as a new subtype of PRCC, is relatively rare in clinic, and can be confirmed by immunophenotype, Diagnosis of OPRCC disease and post-operative management are challenging.so as to clarify the prognosis and survival model of the disease. The pathological and immunophenotypic characteristics of OPRCC were as follows: the tumor boundaries were clear, there was a fibrous envelope, and the tumor cells showed eosinophilic and granular changes under electron microscope. The tumor cells were arranged in a single layer toward the papillary surface. P504S(+) is a sensitive immunomarker. The genetic manifestations of OPRCC are trisomy changes in multiple chromosomes (chromosomes 7, 12, or 17) and loss of the Y chromosome in male patients. Compared with common renal cell carcinoma, OPRCC is relatively less invasive and has a better prognosis, so it is important to regularly review and early follow-up treatment after surgery.

## 2 Case report:1

A 47-year-old male patient was admitted to hospital one week after physical examination for a left kidney tumor. The patient presented with hematuria, accompanied by blood clot, occasional dysuria and urinary tract irritation without obvious inducement or reason one week before, and no lumbago, anorexia, fatigue, emaciation and other systemic symptoms.

1.Color ultrasound of urinary system was conducted on September 2, 2023 in our hospital before admission: echoes of 3.1cm*2.6cm*2.2cm were found in the upper left renal collecting system, the boundary was not clear, and blood flow was visible around the periphery.

2. Lower abdominal Computed Tomography (CT) plain scan + enhancement (conducted on September 6, 2023): Malignant tumor of left kidney (description: circular and slightly low-density shadow in left kidney, progressive uneven enhancement was observed on enhanced scan, about 4.6cm*3.7cm in size), clinical Laboratory test report was generally normal.

The patient was considered to have renal tumor with hematuria. The preliminary diagnosis was left renal malignancy (cT3aNxMx). The clinical stage was set as stage III renal cancer, and the surgical protocol was set as laparoscopic radical resection of left renal cancer.

Pathological diagnosis after operation: (left kidney) combined with immunohistochemistry was consistent with OPRCC. The tumor volume was 3.8cmx3cmx3cm. The tumor invaded the renal pelvis but did not invade the capsule of the kidney. Pathological stage (refer to AJ0C 8th Edition):pT3aNxMx. Immunohistochemical results: Vimentin (+), E - 0 adherin (+), PAX - 8 (+), RCC (+), P504S (+), CD10 (+), EMA (+), CAIX (+) a few, CD117 (-), CK7 (-), TFE3 part (-), 34βE12 (-), FH (+), SDHB(+),Ki-67(10%+).

Pathological stage (refer to AJCC 8th Edition): pT3aNxMx

After surgery, the patient received pabolizumab 200mg (once every three weeks) intravenous drip.

**Figure 1:**
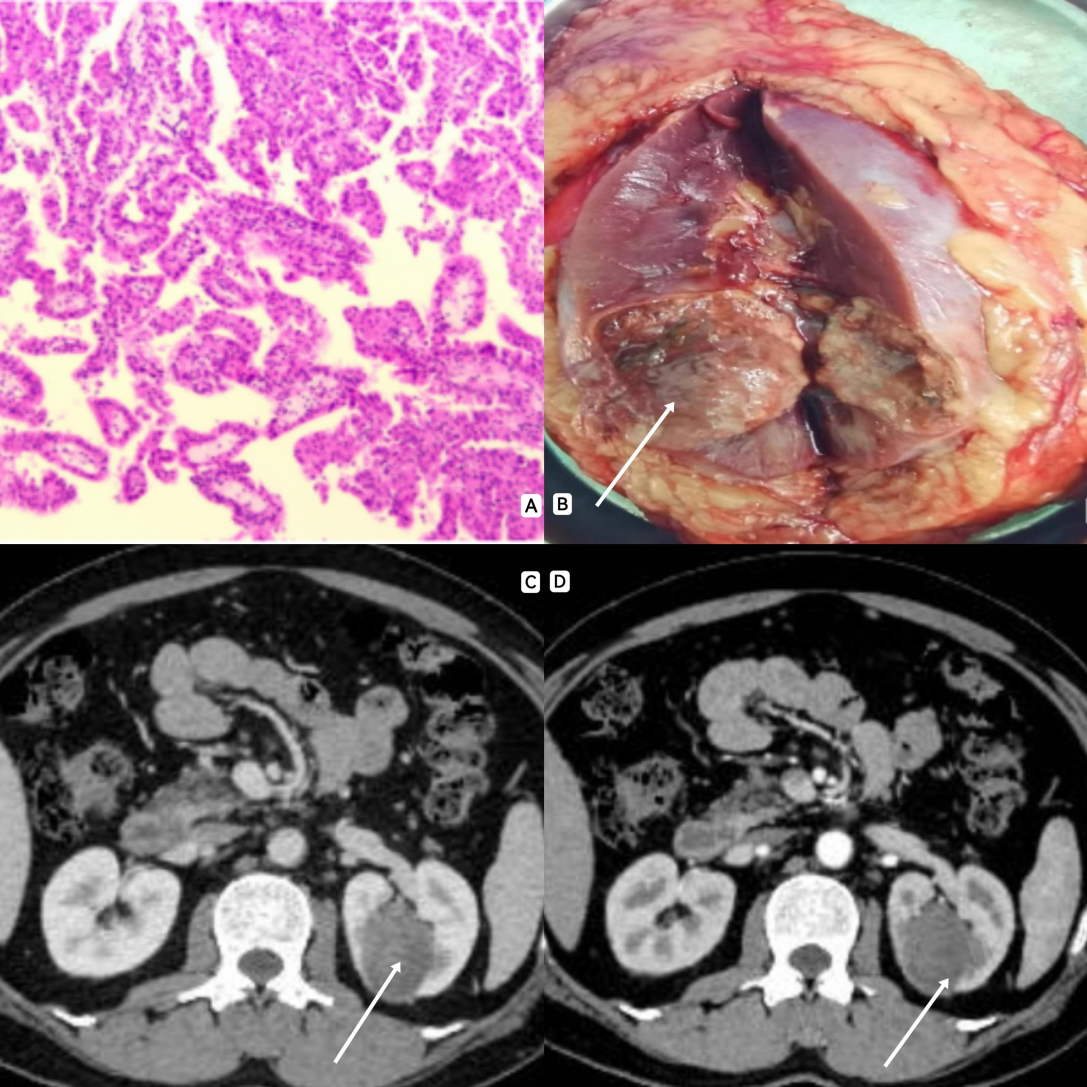
**(A)** A large number of papillary structures were observed under electron microscopy, and the surface of the papillary structure was covered with a large range of single layer of eosinophilic cells, and a strong eosinophilic cytoplasmic envelope was observed in the axis of the papillary structure. **(B)**: The area indicated by the arrow shows a renal tumor, about 4cm*3cm in size. **(C, D)**: CT often showed solid mass with lack of blood supply, clear boundary and mild enhancement.

Regular follow-up for 15 months., no signs of tumor recurrence and metastasis (**Figure 1**).

## 3 Case report:2

A 74-year-old male patient was admitted to the hospital 2 days after physical examination for a tumor in his right kidney. The patient underwent a physical examination at a local hospital 2 days ago and underwent abdominal CT: multiple right kidney Spaces with different nature. After admission, further lower abdominal CT plain scan + intensification was performed: the possibility of right renal cell carcinoma with perirenal fat sac metastasis was considered.

History of hypertension, old cerebral infarction, type 2 diabetes

Surgical history: Partial renal surgery (open) was performed in our hospital for right renal eosinophilic tumor 10 years ago. Surgery for gallstones 30 years ago;

Physical examination showed a 15cm surgical scar on the right waist.

Preliminary diagnosis: Right renal malignancy (cT3aNxMx) with stage III clinical stage. Laparoscopic radical resection of right renal carcinoma (conversion to open operation) was the surgical protocol.

Postoperative pathological diagnosis: (right kidney and tumor) matched OPRCC in combination with immunohistochemistry. The tumor was accompanied by bleeding, degeneration and necrosis, with a total volume of 10cmx7cmx3.5cm. The renal pelvis, renal capsule and perirenal fat were not involved, peripheral adrenal tissue was not involved, and intracavitary tumor and nerve invasion were not observed. The incision margin of ureter and blood vessel was clean: no tumor metastasis was found in hilar lymph nodes (0/5).

Pathological stage (refer to AJ0C 8th Edition):pT3aN0Mx

Immunohistochemical results: P504S (+), CK7 (-), CK20 (-), CD15 (a few +), RCC (+), CD10 (part + Vimentin (+), PAX - 8 + (part), EMA (in +), CD117 focal (+), Ki - 67 index (5%),Colloidal iron staining (-).

After surgery, the patient received pabolizumab 200mg (once every three weeks) intravenous drip.

**Figure 2:**
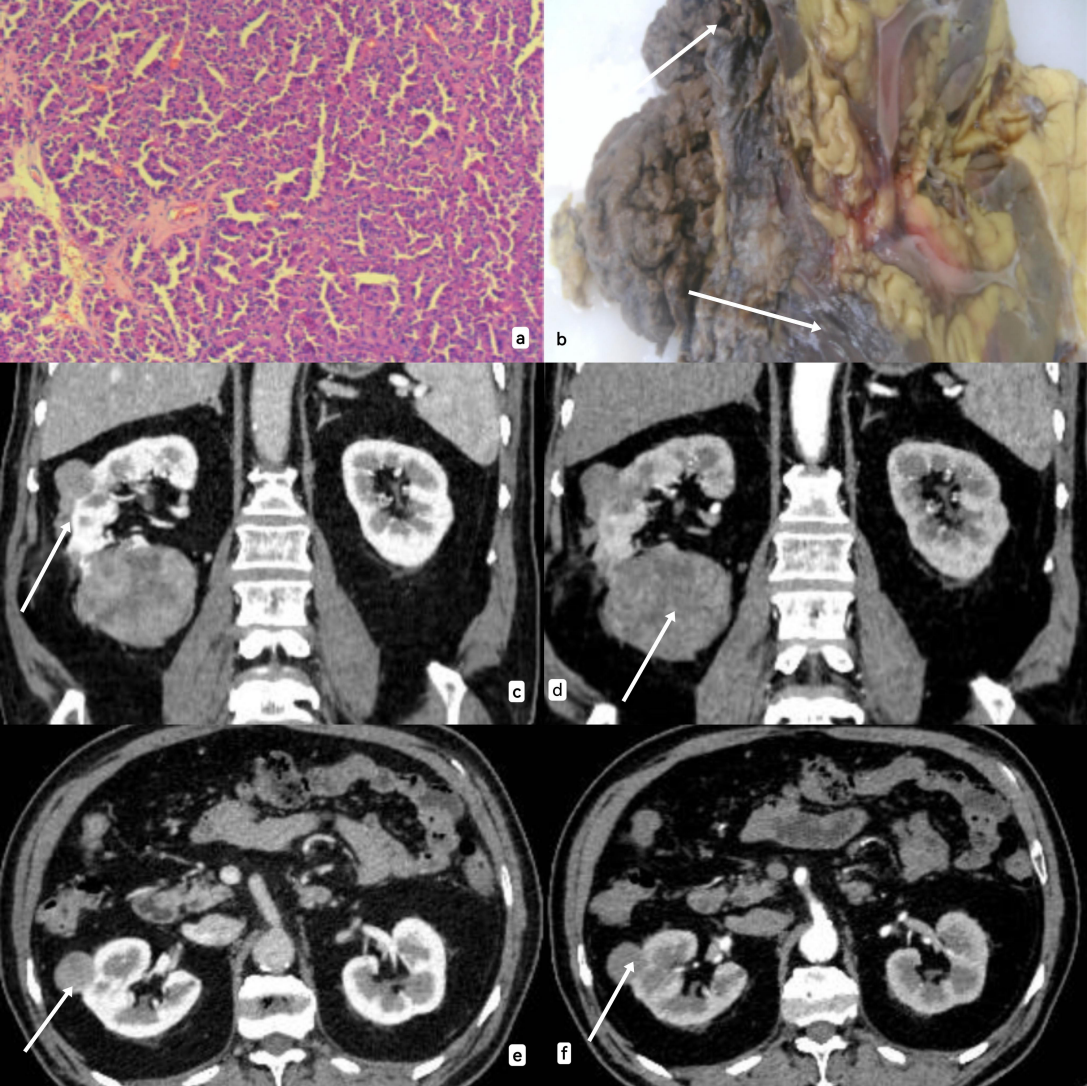
**(A)** Under electron microscope, tumor cells showed cytoplasmic eosinophilic, low nuclear grade, and polar arrangement toward the papillary surface. **(B)**: The area pointed by the arrow is the solid specimen of the kidney tumor (due to the open operation during the operation, the specimen display was not clear). **(C–F)**: CT information of the tumor was shown from the sagittal plane and cross section respectively, and CT showed a solid mass lacking blood supply with clear boundaries and mild enhancement.

Regular follow-up for 13 months. no signs of tumor recurrence and metastasis (**Figure 2**).

## 4 Discussion

Oncocytic papillary renal cell carcinoma (OPRCC)is a relatively rare subtype of papillary renal cell carcinoma. Recently, the author searched the data of 85 patients with OPRCC in 14 articles through Pubmed, CNKI, Wanfang and VIP databases, etc. According to the currently searchable reports (**Table 1**), OPRCC mostly occurred in the middle-aged and elderly, and was mostly male, accounting for 68.2%. The mean age was 64.6 years. As early as 2005, Lefevre et al. (1) reported 10 cases of PRCC with papillary structure, eosinophilic cytoplasm, and low-grade nonoverlapping nuclei, and further compared them with PRCC types I and II. The histological characteristics of OPRCC were characterized by eosinophilic cytoplasmic rich cytoplasm. Thus, OPRCC was identified as another new subtype of PRCC. The classification of PRCC is of great significance for judging the prognosis of patients and selecting appropriate treatment plans. The histopathologic characteristics of papillary renal cell carcinoma type I are that the tumor cells are rich in low-grade nuclei and the papillary structure is covered by a single layer. Histopathologic characteristics of papillary renal cell carcinoma type II In addition to the type I PRCC characteristics, the cytoplasm of the tumor cells is rich in eosinophilic cells. Although the 5th edition of WHO Classification of renal cell carcinoma no longer distinguishes PRCC type I from type II, the distinction between type I and type II is of great guiding significance from the perspective of clinicians observing the disease progression and prognosis of patients. The prognosis is better than type II. Relevant studies have shown that type I and type II PRCC have statistically significant differences in biological behavior and prognosis, with 5-year survival rates of 92% and 44% respectively (2). OPRCC, as a new subtype of PRCC, is similar to but different from PRCC type I and type II in morphology, immunophenotype and genetics. Compared with clear cell renal cell carcinoma (CCRCC), OPRCC is less invasive and has a better prognosis. However, for locally advanced or advanced OPRCC, aggressive posterior line treatment is necessary to improve prognosis survival.

**Table 1:** Literature review results of 85 patients with OPRCC.

No	Docu	Case	Median age (years)	Ratio of male to female	Radical resection	Renal preservation operation	Median follow-up time (months)	Progress
1	Okada A et al. (16)	1	81	0:1	1		12	Come to no avail
2	Lefèvre M et al. (1)	10	71	10:0	10		62	Come to no avail
3	Hes O et al. (7)	12	67	10:2	12		61.5	There was no progression in 11 cases and local recurrence plus brain metastasis in 1 case 2 years later
4	Park BH et al. (3)	7	67	3:4	2	5	37	Come to no avail
5	Xia Q-Y et al. (4)	6	56.8	5:1	6	0	84	Come to no avail
6	Han G et al. (17)	14	64	7:7	14	0	37	There was no progression in 36 patients, and 1 patient died of bone metastasis 8 months after surgery
7	Ürge T et al. (18)	12	67.5	8:4	7	5	35	Come to no avail
8	N Masuzawa et al. (19)	1	78	1:0	1	0	16	Come to no avail
9	Kim NR et al. (20)	1	52	1:0	1	0	34	Come to no avail
10	Kunju LP et al. (5)	7	57	5:2	2	5	22	Six cases did not progress and one died of pancreatic cancer
11	XIA Qiu-yuan,et al. (21)	6	57	5:1	6	0	84	There was no progress in 4 cases and no follow-up in 2 cases
12	NIANYe-qi,et al. (22)	1	48	0:1	1	0	6	Come to no avail
13	LIU Dan,et al. (23)	5	71	2:3	4	1	41	Come to no avail
14	NIU Xiao,et al. (24)	2	66.5	1:1	1	1	11	Come to no avail

OPRCC has no typical clinical symptoms, most patients come to the hospital for medical treatment when kidney tumors are found during physical examination, and some patients come to the hospital for hematuria or low back pain, so the diagnosis of OPRCC needs to rely on histopathological examination. Histological studies have shown that OPRCC’s tumor cells are derived from proximal tubule epithelial cells, which are homologous to papillary renal cell carcinoma, whereas renal eosinophil tumor (RO) is derived from distal tubule epithelial cells, unlike RO. The diagnosis of OPRCC mainly depends on pathological and immunohistochemical manifestations. The pathological tissue of OPRCC showed extensive papillary structure under electron microscope, and the surface of the papillary structure was covered by a large range of single layer of eosinophilic cells, and the cells showed small and round nuclei, no obvious atypia and low nuclear grade. In the axis of the papillary structure, there are a variety of foamy giant cell cytoplasmic envelope which is strongly eosinophilic. In combination with these diseases reported by several researchers (2–5), the author concluded that the immunophenotypic characteristics of OPRCC were positive for RCC (+) and AMACR (P504S) (strongly expressed in the papillary region and weakly expressed in the eosinophilic region), and low proliferative indices of Vimentin (+), CK10 (+/-) and Ki-67 (5%-10% +). CD15, CD17 (+/-) (+/-), CK7 (+/-), CK19 were (+/-), CD117 (+/-). AMACR (P504S), as a specific and sensitive marker of PRCC, is also highly expressed in OPRCC, while its expression is relatively small and weak in the immunohistochemistry of other renal tumors. Combined with its immunohistochemical characteristics, AMACR can be clearly diagnosed.

Although the immunophenotypes of PRCC type I and type II are different, the PRCC immunophenotypes of type I usually show positive diffuse immunostaining for AMACR (P504S), vimentin, CK7, and EMA, while type II PRCC usually shows positive immunostaining for AMACR (P504S), vimentin, and CD10. Gobbo S et al. (6) concluded that the immunophenotype CD10 expression rate and CK7 and EMA expression rate of OPRCC are higher, and the immunophenotype is more similar to type II PRCC, confirming that OPRCC is an immunophenotype tumor group that is more similar to type II PRCC. Therefore, the prognosis of the disease needs high attention. With the help of IHC, the diagnosis of OPRCC can be subdivided from PRCC to make a more accurate diagnosis, and at the same time, patients and their close families can be informed that the prognosis of OPRCC is relatively poor, and regular follow-up after surgery is needed, which is crucial.

Some researchers have concluded that the genetic manifestations of OPRCC are mainly trisomy changes in multiple chromosomes (chromosomes 7, 12 or 17), and loss of the Y chromosome in male patients. When He O et al. (7)conducted FISH detection on 12 OPRCC patients, 7 patients had triploid on chromosome 7, 3 male patients had Y chromosome deletion, and 8 patients had triploid on chromosome 17. Gobbo S et al. (6)found that chromosomes 3p, 11q, and 17 of OPRCC patients were triploid, while chromosome 4q was missing. If further spatial transcriptome sequencing was conducted on pathological sections, further research was focused on the related factors of chromosome 7, 12, 17, Y and other chromosome deficiency, which may be a breakthrough point for the treatment of this disease from the genetic level.

Based on the reports of these two cases and the relevant imaging data reviewed recently, the imaging characteristics of OPRCC were summarized: 1. CT characteristics: The CT characteristics of OPRCC were consistent with the imaging findings of PRCC, which often showed unilateral single tumor on CT, and occasionally showed multiple circular or circular tumors on both sides. CT plain scan showed solid mass with uniform density (mostly medium density or low density), with clear boundaries, usually occurring at the dermatomedullary junction, often protruding from the kidney surface, most of them had obvious envelope, prone to bleeding, necrosis, cystic change, and occasionally low-density necrotic area or punctal calcification could be seen inside. CT enhanced scan showed solid mass with lack of blood supply. It is characterized by a slow rise (mild strengthening in the medullary phase, lower than the renal cortex, an increasing trend in the medullary phase, generally mild strengthening) (8, 9).2. Ultrasonic characteristics: The ultrasound examination of OPRCC generally presents as solid masses with equal-echo or low-echo, with poorly defined boundaries and regular morphology, which generally protrudes outside the renal capsule, and surrounding blood flow signals can be seen around the tumor. 3. Magnetic resonance imaging (MRI) features: the MRI findings of OPRCC showed that most of the tumors were single solid or solid cystic masses, with different sizes, regular shapes and roundness. Most tumors were exogenous growth in the renal cortex, while a few tumors were endogenous and confined to the medulla of the renal skin. T1WI showed equal or slightly high signal, and there was no significant difference from the inverse phase signal, T2WI showed low signal, DWI mostly showed slightly high or high signal, occasionally low signal, ADC showed low signal lesion, lower than the renal cortex ADC signal, and a few showed slightly low signal (10).

Because OPRCC is characterized by papillary structure, abundant eosinophilic plasma, and chromosomal triploid changes, Clinical diagnosis needs to be differentiated from clear renal cell carcinoma eosinophilic renal cell carcinoma, chromophobe renal cell carcinoma, type II PRCC and RO, MiT family translocation renal cell carcinoma, fumarate hydratase deficient renal cell carcinoma (FH-d RCC) and other renal cell carcinoma subtypes. 1. OPRCC can be easily distinguished from eosinophilic subtypes of clear renal cell carcinoma and chromophobe renal cell carcinoma due to its unique clinicopathological features and genetic characteristics (persistent papillary structure, P504S+ and trisomy of chromosome 7, 12 or 17, Y staining deletion). 2. The pathological tissue of RO showed extensive eosinophilic and focal solid structure under electron microscope, with less papillary cell structure, and the immune appearance was CD117 immunostaining positive and P504S-. Due to the radial scar in the central part of the tumor in some patients, the central “star-shaped scar” found by CT or MRI examination can also help diagnosis (2, 11). 3. The pathogenic mechanism of MiT family translocation renal carcinoma is that chromosomal translocation forms the corresponding mosaic-protein resulting in overexpression of transcription factors EB and E3, which promotes tumor formation. The diagnosis can be made clearly through FISH detection or second-generation gene sequencing. Papillary growth, large nucleoli, acidophilic or transparent cytoplasm, with a large number of sandy bodies. 4. Fh-d RCC is a clinical manifestation of skin and/or uterine leiomyoma and renal cell carcinoma caused by FH gene mutation (system/germ line), and has familial heritability. Typical FH-D RCC tumors under electron microscopy have eosinophilic cytoplasm, significant nuclear atypia, large nucleolar virus-like inclusion bodies in the nucleus, and obvious perinuclear halo. Typical immunohistochemistry (IHC) results were loss of FH expression while next generation sequencing (NCS) diagnoses were FH gene mutations (12). Therefore, the diagnosis of the disease can be confirmed by IHC staining and NGS. 5.VHL syndrome is a dominant familial genetic disease, with lesions covering the central nervous system, retinal hemangioblastoma, visceral tumors (especially renal cancer, adrenal phaochromocytoma), pancreatic cysts, multiple renal cysts, etc. The diagnosis of this disease can be confirmed by detecting the mutation of VHL gene.

Combined with the above diagnosis and treatment of two patients, the experience of OPRCC operation and follow-up treatment was summarized and reviewed. At present, there have not been a large number of clinical studies on the surgical methods of OPRCC. According to the 14 articles retrieved in **Table 1**, a total of 85 patients with OPRCC, it can be found that both radical surgery and renal preservation surgery programs exist, and the median follow-up time was from June to 84 months. According to the retrieved data, except 2 patients lost follow-up, only 3 patients with OPRCC died. It can be seen that the clinical progression of OPRCC disease is slow and the prognosis is good. Therefore, accurate diagnosis of PRCC subtype is of great significance for guiding the treatment of patients and predicting their prognosis.

For patients with limited early OPRCC, regular follow-up can be done after surgery. After entering the era of targeting and immunity, rich experience has been accumulated in the selection of drugs for the treatment of locally advanced/advanced OPRCC combined with multi-center studies. Although there are few reports on the treatment of OPRCC diseases, some researchers (13, 14) have reported that mesenchymal-epithelial transition factor (MET), as a receptor for hepatocyte growth factor (HGF), belongs to the tyrosine kinase-TK family, and the HGF/MET signaling pathway is involved in the growth, survival, proliferation, differentiation and cell migration of tumor cells in OPRCC diseases. Overexpression of MET gene can be found in chromosomes 7 and 17 of OPRCC patients, so TKI drugs are effective against OPRCC; Gilbert JA et al. (15)found that the MET-Tki drug Savolitinib was effective in treating advanced PRCC patients with MET changes. With the advent of various targeted drugs, patients with advanced OPRCC can be stratified according to International Metastatic Renalcell carcinoma Database Consortium regulations(IMDC), and it can be inferred that first-line targeted drugs, such as sorafenib, Sunitinib, Pezopanib, and cbottinib, are effective.

The two patients were pathologically confirmed to have stage III renal cancer (pT3N1M0) after surgery. Currently, there is no unified treatment plan for follow-up treatment of patients with locally progressive OPRCC. Clinically, OPRCC patients differ greatly in their responses to targeted and immune drugs, and some researchers suggest spatial transcriptome gene sequencing to guide the precise selection of drugs. For individual therapy, the idea is worth thinking about. The two patients were treated with pabolizumab immune drug -200mg/3 weeks after surgery, and were followed up for 15 and 13 months, respectively. No significant signs of progress were found in both patients.

## 5 Conclusion

OPRCC is a rare subtype of PRCC, which is extremely rare clinically, and its morphology, immunophenotype, genetics and prognosis are still uncertain. IHC staining and NGS are needed for definitive diagnosis. Meanwhile, spatial transcriptome gene sequencing can further clarify the problem of OPRCC gene deficiency to guide individualized patient treatment. The combination of targeted and immune checkpoint inhibitors may be a better treatment strategy and should be promoted.

## Data Availability

The original contributions presented in the study are included in the article/supplementary material. Further inquiries can be directed to the corresponding authors.
